# Determinants of unmet need for family planning among women in Urban Cameroon: a cross sectional survey in the Biyem-Assi Health District, Yaoundé

**DOI:** 10.1186/s12905-016-0283-9

**Published:** 2016-01-20

**Authors:** Atem Bethel Ajong, Philip Nana Njotang, Martin Ndinakie Yakum, Marie José Essi, Felix Essiben, Filbert Eko Eko, Bruno Kenfack, Enow Robinson Mbu

**Affiliations:** 1 Department of Obstetrics and Gynaecology, Faculty of Medicine and Biomedical Sciences, University of Yaoundé I, Yaoundé, Cameroon; 2 Obstetrics and Gynaecology unit, Yaoundé Central Hospital, Higher Institute of Health Technologies, Yaoundé, Cameroon; 3 Meilleur Accès aux soins de Santé, Yaoundé, Cameroon; 4 Department of Public Health, Faculty of Medicine and Biomedical Sciences, University of Yaoundé I, Yaoundé, Cameroon; 5 Department of Biomedical Sciences, Faculty of Science, University of Dschang, Dschang, Cameroon; 6 Directorate of Family Health, Ministry of Public Health, Yaoundé, Cameroon

**Keywords:** Unmet need, Determinant, Contraception, Family planning, Biyem-Assi

## Abstract

**Background:**

With the unacceptably high level of unmet need for family planning in Sub-Saharan Africa, reducing unmet need is paramount in the fight against the high levels of induced abortions, maternal and neonatal morbi-mortality. A clear understanding of the determinants of unmet need for family planning is indispensable in this light. The objective of this study was to determine the prevalence of unmet need for family planning in Urban Cameroon while identifying major determinants of unmet need among women in a union in Urban Cameroon.

**Methods:**

A community based cross sectional study was conducted from March 2015 to April 2015 during which 370 women in a union were recruited using cluster multistep sampling in the Biyem-Assi Health District, Yaounde. Data were collected using a pretested and validated questionnaire. Proportions and their 95 % confidence intervals were calculated with the Westoff/DHS method used to estimate unmet need for family planning and the odds ratio used as measure of association with statistical significant threshold set at *p*-value ≤ 0.05.

**Results:**

Of the 370 eligible women included, the mean age was 29.9 ± 6.8 years, and 61.1 % were married. The prevalence of unmet need for family planning was 20.4 (16.4-24.8)% with 14.2 (11.2-18.7)% having an unmet need for spacing and 6.2 (3.6-8.7)% an unmet need for limiting. Husband’s approval of contraception had a statistically significant protective association with unmet need (AOR = 0.52 [0.30-0.92], *p* = 0.023), and discussion about family planning within the couple had a highly statistically significant protective association with unmet need (AOR = 0.39 [0.21-0.69], *p* = 0.001). The major reason for non-use of contraception among women with unmet need was the fear of side effects.

**Conclusion:**

The prevalence of unmet need of family planning among women in the Biyem-Assi Health District remains high. Husband’s approval of contraception and couples’ discussion about family planning are two major factors to be considered when planning interventions to reduce unmet need for family planning. Family planning activities focused on couples or including men could be useful in reducing the rate of unmet need in Cameroon.

## Background and rationale

Most of the developing world is far from meeting the targets set by the Millennium Development Goals (MDG) on maternal mortality and access to reproductive health services (reducing maternal mortality by 75 % and assuring a universal access to reproductive health services come 2015) [1, 2]. In most African countries, maternal mortality has shown instead rising trends and for some countries which have made progress, the target is far from being met [1, 2]. Cameroon is not an exception; in fact, according to different national demographic and health surveys, even with the rising prevalence of modern contraception, maternal mortality has shown rising trends over the years: 551 (in 1998), 669 (in 2004), and 782 (in 2011) maternal deaths per 100.000 live births [3]. Notwithstanding, between 1990 and 2013, Sub-Saharan Africa has experienced a significant reduction in maternal mortality rates (49 % reduction) but still far from the MDG 5 target [1, 2, 4]. Among diverse indicators of attainment of these goals were prevalence of contraception and the unmet need for family planning of which the post-2015 development agenda (Sustainable Development Goals) still has these two indicators [1, 2].

Unmet need for family planning in Africa is still highest [2, 5] and more precisely in Cameroon it is still unacceptably high [3, 6]. This shows great discrepancy between the fertility desires of women in this part of the world and their present contraceptive practices. Meeting contraceptive needs among women will go a long way in drastically reducing maternal morbi-mortality [4, 5, 7, 8] and the high rate of induced abortion in Cameroon [9, 10]. With the developing world accounting for about 99 % of maternal deaths in the world [7], Africa and more precisely Cameroon is a very important target for interventions aimed at increasing potential contraceptive demand by reducing unmet need for family planning.

According to literature, diverse factors including age, marital status, level of education, religion, occupation, and household wealth have been found to be significantly associated to unmet need for family planning [3, 5, 6]. Misconceptions and concern of the health risk associated to these methods influence in a significant way contraceptive use and therefore keep unmet need high. Understanding the different determinants of unmet need for family planning and the major reasons of non-use among these women will go a long way to help policy makers plan interventions aimed at reducing unmet need for family planning. In Cameroon, literature on this subject matter is still sparse and the determinants of unmet need among women in urban Cameroon have not been clearly elucidated. In addition, little or no studies have clearly identified the above mention determinants to be true in the Urban Cameroon context.

This study was therefore aimed at determining the prevalence of unmet need for family planning, identifying determining factors of unmet need for family planning among women in a union, and reasons for non-use of contraception among women with unmet need for family planning in the Biyem-Assi Health District.

## Methods

### Study design

A cross sectional community based study was conducted targeting sexually active women of child bearing age in a union living in the Biyem-Assi Health District of Yaoundé. The selection of participants involved a multi-step cluster sampling technique. The data were collected using a pretested and validated questionnaire and analysed using the statistical software Epi Info version 3.5.4.

### Setting

The Centre region of Cameroon (with Yaoundé as capital city) is made up of 31 Health Districts with the Biyem-Assi Health District being the largest and most populated in Yaoundé. It is an urban Health District extending over a surface area of 22 km^2^ and made of four Health Areas (Melen, Mendong, Biyem-Assi and Mvog-Betsi). It has a cosmopolitan population with over 81 000 women of childbearing age in 2015. One of the highest prevalence of contraceptive use during the 2011 demographic and health survey was reported in Yaoundé [3] and given that the Biyem-Assi Health District is the largest in Yaoundé with a cosmopolitan population, it was chosen chosen for this study. Data was collected from March 2015 to April 2015.

### Participants

- ➢ Eligibility criteria
  All sexually active women in a union aged 15 to 49 years living in the Biyem-Assi Health District (according to the 2011 demographic and health survey, the highest level of unmet need was registered among women in a union) [3]
- ➢ Exclusion criteria
  - ✓ Women not yet sexually active aged 15-49 years.
  - ✓ Visitors to the Biyem-Assi Health District.
  - ✓ Women of childbearing age who were mentally incapacitated.

### Sample size and sampling

The minimum sample size of the study was calculated using the following parameters: the expected proportion of women with unmet need for family planning, the absolute precision required on either sides of the proportion, threshold of error at 5 % and a cluster effect of 2. A total of 370 women in a union of childbearing age were included in the study. All four health areas (Mendong, Melen, Mvog-Betsi, and Biyem-Assi) of the Biyem-Assi Health District were included and a two-step cluster sampling applied. The total population of the Health District was divided into 70 geographical clusters and 50 randomly selected while respecting the number of clusters per health area. In each selected cluster, all the major road junctions were identified and one selected randomly. In a selected junction, a direction was chosen randomly by tossing a plastic bottle and following the direction of the head. Once on the chosen street, only households on the left hand side of the interviewer were considered. All households in which we were received were included successively in a chronological manner. In each household, all eligible and consenting participants were enrolled into the study.

When the protocol, the questionnaire and informed consent form were developed, the data collection tools were pretested and validated. Interviewers were recruited and trained on the consenting and data collection procedures and then divided into four (4) teams each headed by a well-trained field supervisor. Upon identification of eligible participants, an informed consent form was administered to the participants. For those who accepted to participate in the study, a questionnaire was administered to them face to face. Their general characteristics, Socio-demographic, economic variables, obstetric history, and information on their contraceptive practices were collected.

### Bias

Given that the populations in the four health areas were all urban and that their characteristics were not very different, a multistep cluster sampling was used to limit the selection bias. To limit lie bias, When a woman accepted to participate, she was administered the questionnaire alone and away from those who did not make up part of the research team. A two-day training session was organised during which the principal investigator presented the questionnaire and trained the interviewers on the consenting and data collection procedure to limit measurement bias.

### Data management and analysis

**Fig. 1:**
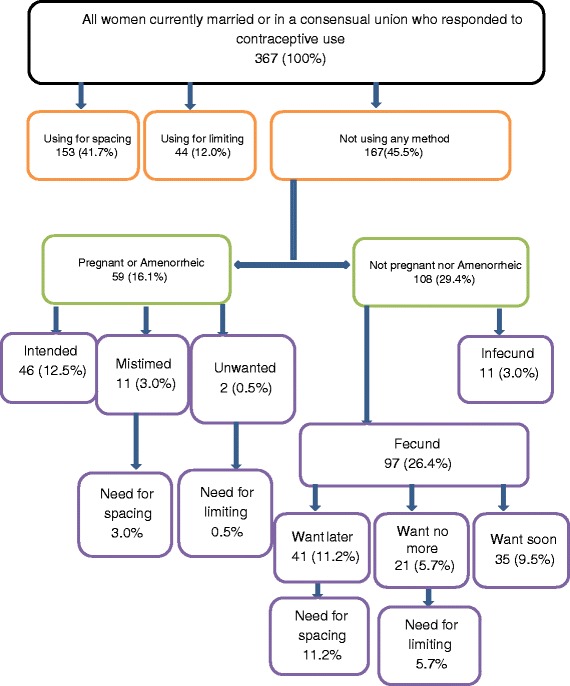
Unmet need for family planning

All the filled questionnaires were verified and validated by the field supervisor. These questionnaires were then transferred to the principal investigator (PI) for conservation. The data capturing or entry sheet was developed using the statistical software Epi Info version 3.5.4 software. The data from the validated questionnaires was then double-entered, compared, cleaned and analyzed. Major analyses included calculation of frequency and their confidence intervals at 95 % (for qualitative variables such as marital status, level of education, occupation,… etc.), and means (for quantitative variables such as age of participant, number of years of cohabitation). We used the “Westoff/Demographic and Health Survey method” of measuring unmet need, also known as the “core definition method” [11], (see Fig. 1). The steps were as follows;

- ✓ Step 1: Contraceptive Use Status: We considered all women in a union of reproductive age; from these we determined the percentage not using contraception.
- ✓ Step 2: Pregnancy and Amenorrheic Status: From the group selected in step 1 above, we divided them into two groups (pregnant or amenorrheic, and the percentage of those not pregnant or not amenorrheic).
- ✓ Step 3: Considering the wantedness of pregnancy and fecundity status from the two identified groups in step 2, the three other percentages were developed (pregnancy mistimed, pregnancy unwanted, fecund).
- ✓ Step 4: For the fecund group identified in step 3 above, we considered future fertility intentions. And calculate the percentages of those who want to postpone childbearing (want later/spacers) and those who want to limit (want no more children/limiters).
- ✓ Step 5: Considering again the groups identified in step 3 (pregnancy mistimed, pregnancy unwanted) and 4 (proportion among those fecund who want childbearing later, and want no more) above, the percentage who have unmet need for spacing and limiting, respectively were calculated. The sum of unmet need for spacing and limiting gave us the final unmet need for family planning.

The potential demand for contraception was then calculated as the percentage of women presently in use of contraception or who had an unmet need for family planning excluding women with unmet need who were pregnant or amenorrheic. The proportion of current contraceptive users among those with a potential demand for contraception was then calculated giving us the proportion of the potential demand currently satisfied.

Factors associated to unmet need for family planning were determined by calculating Odds Ratios (OR) at 95 % Confidence Interval (CI) with the significant threshold set at *p*-value ≤0.05. The statistical test used during univariate analysis was chi square but multiple logistic regression was used during multivariate analysis. The following factors were tested for association: level of education of the woman, Religion, number of children alive, total number of pregnancies, partner’s approval of contraception, and discussion of family planning within the couple. Potential confounders controlled were level of education of the partner, estimated monthly revenue, age of the woman, and occupation. The selection of these factors as potential confounders was based on literature identifying them as such [3, 5, 6]. The results were presented in figures and tables.

## Operational terms

- ➢ **Woman with an unmet need for Family planning:** This refers to any woman not known to be infertile, with no desire of childbearing in her life, or desires to postpone childbearing for at least two years and is not using a contraceptive method (be it traditional or modern). Or who has her present pregnancy or has had her last pregnancy unplanned.
- ➢ **Infertile woman:** A woman who reported to be infertile, or who has undergone hysterectomy, bilateral tubal ligation, or bilateral salpingectomy or has a non-gestational amenorrhoea for more than 1 year.
- ➢ **Woman of child bearing age**: Any woman between 15 and 49 years of age
- ➢ **Sexually active woman**: a woman who had her first sexual contact at least three months before onset of the study
- ➢ **A woman in a consensual union**: A woman living with the partner under the same roof as married without formal ratification by the law for at least six (6) months. Also said to be in a “free union”.
- ➢ **Woman in a union**: A woman who is either in a free union or is married.

## Ethical considerations

Participant’s confidentiality and autonomy were respected during this study. The participants were well informed and were given enough time to freely decide to participate or not. Every eligible participant who was ready to participate signed an informed consent form giving us the go ahead to administer the questionnaire. Above all, an ethical clearance was obtained from the institutional ethical review board of the Faculty of Medicine and Biomedical Sciences of the University of Yaoundé I and an administrative authorisation obtained from the Biyem-Assi District Health Service.

## Results

Out of the 396 eligible participants contacted for the study, 26 refused to participate giving a non-response rate of 6.6 %. A total of 370 women who met inclusion criteria were included in our study. Their ages ranged from 18 to 49 years with a mean age of 29.9 ± 6.8 years. A good proportion was married (61.1 %) while the rest were in a consensual union. The mean number of years of cohabitation among married women (8.9 ± 7.8 years) was about doubled that among women in a consensual union (4.9 ± 4.3 years). About half (54.3 %) reported an approximate monthly revenue of less than 50 000FCFA (80.75 US dollars). About 4 out of every 5 (79.5 %) women had acquired at least a secondary education and the population was dominated by Christians (95.3 %) of different denominations and the rest Muslims. Of the 367 who responded to whether they had used contraception or not, 328(89.1 %) had used at least one contraceptive method from their first sexual contact.

### Unmet need for family planning

Figure 1 shows the different steps used in evaluating the unmet need for family planning and associated results. It was estimated that 20.4 [16.4-24.8]% of the women in a union had an unmet need for family planning with 14.2(11.2-18.7)% having an unmet need for spacing and 6.2(3.6-8.7)% having an unmet need for limiting. The potential demand for contraception (the percentage of women presently in use of contraception or who had an unmet need for family planning excluding women with unmet need who were pregnant or amenorrheic) among women in a union was at 70.6 % (41.7 % +12.0 % + 11.2 % + 5.7 %) with a 52.9 % (41.7 % + 11.2 %) demand for spacing and 17.7 % (12.0 % + 5.7 %) demand for limiting. The proportion of the potential demand currently satisfied was 76.1 %. (see Fig. 1).

### Determinants of unmet need for family planning

Table 1 shows the evaluation of the determinants of unmet need for family planning both in univariate and multivariate analysis. Following univariate analysis, unmet need for family planning was significantly associated to total number of pregnancies, number of children alive, approval of contraception by the partner and discussion of family planning within the couple. There was some degree of association though statistically insignificant between unmet need for family planning and level of education, religion, and number of years of cohabitation.

**Table 1:** Determinants of unmet need for family planning

Factors	Univariate analysis			Multivariate analysis		
	OR	CI 95 %	*p*-value	Adj OR	Adj CI 95 %	*p*-value
Muslim (Y/N)	1.68	0.57-4.94	0.342	1.83	061-5.44	0.279
Number of years of cohabitation > 5 (Y/N)	1.18	064-2.19	0.591	1.05	051-2.17	0.901
Level of education above primary (Y/N)	1.16	0.61-2.21	0.653	1.74	0.82-3.70	0.150
Total number of pregnancy > 3 (Y/N)	1.84	1.07-3.17	0.027*	1.82	0.96-3.46	0.066
Number of children alive > 5	2.78	1.02-7.60	0.046*	2.77	0.93-8.23	0.068
Does your partner approve of contraception? (Y/N)	0.53	0.31-0.92	0.025*	0.52	0.30-0.92	0.023*
Discussion of family planning within the couple. (Y/N)	0.40	0.25-0.70	0.002*	0.39	0.21-0.69	0.001*

Y/N = Yes/No, OR = Odds Ratio, Adj OR = Adjusted Odds Ratio, CI = Confidence Interval, Adj CI: Adjusted Confidence interval, * Statistically significant (*p*-value ≤ 0.05)

Following a multivariate analysis with possible confounders, a statistically significant association was noted between unmet need and partner’s approval of contraception [AOR(Adjusted Odds Ratio) = 0.52 [0.30-0.92] *p* = 0.023] and a very high statistically significant association between unmet need and discussion of family planning within the couple (AOR = 0.39 [0.21-0.69], *p* = 0.001).

### Reasons for non-use of contraception among women with unmet need for family planning

Table 2 presents reasons for non-use of contraception among women with unmet need for family planning. Fear of side effects (37.3 %), partner’s disapproval of contraception (20.0 %) and religious beliefs (13.3 %) are major reasons women with an unmet need for family planning do not used contraception.

**Table 2:** Reasons for non-use of contraception among women with unmet need for family planning

Reason for unmet need	Frequency	Percentage (*n* = 75)
Fear of side effects	28	37.3
My partner does not approve contraception	15	20.0
I don’t have sex frequently	8	10.7
Lack of access	5	6.7
My religion is against	10	13.3
Others	9	12.0

## Discussion

This paper evaluated the magnitude of unmet need for family planning among women in Urban Cameroon as well as identified major determining factors for unmet need for family planning in this population. The prevalence of unmet need for family planning was 20.4 (16.4-24.8)%. Husband’s approval of contraception had a statistically significant protective association with unmet need (AOR = 0.52[0.30-0.92], *p* = 0.023), and discussion about family planning within the couple had a highly statistically significant protective association with unmet need (AOR = 0.39[0.21-0.69], *p* = 0.001). The major reason for non-use of contraception among women with unmet need was the fear of side effects.

Unmet need for family planning among these women was still very high (20.4 % with 14.2 % for spacing and 6.2 % for limiting) with a potential demand for family planning among these women of 70.6 % (52.0 % for spacing and 17.7 % for limiting). Only 76.1 % of this demand is satisfied as of now. This means that 1 out of every 5 women had an unmet need for family planning. Similar results have been registered in many other studies: in 2011, the National Demographic and Health Survey in Cameroon reported a prevalence of unmet need in Yaounde of 24.4 % (17.5 % for spacing and 7 % for limiting) with a potential demand for contraception of 61.7 % and a percentage satisfaction of 60.4 % [3]. In 2012, the prevalence of unmet need for family planning among married women in Rural India was 23.9 % [12]. The discrepancy in the results could be explained by sample variation, the highly urbanized nature of the Biyem-Assi Health District which has increased cost of living imposing a check on the family sizes, and efforts put in place by the Cameroon government to improve on the family planning delivery services all of which have contributed to increase the potential demand for family planning over the years.

Our result was however very low compared to that reported in Urban Ethiopia (prevalence of unmet need for family planning was 52.4 %) [5] and in Uganda (unmet need of 34.2 %) [13]. This could be explained by the fact that the sociocultural beliefs of the these two settings are different from what we have in Urban Cameroon. The perception of contraception, attitudes towards contraception and method accessibility in Cameroon are likely to be more positive compared to these other settings. With a potential demand of contraception among women in a union at 70.6 %, and only 76.1 % of their potential contraceptive needs satisfied, up to about a quarter of their potential contraceptive demands are still to be looked into and resolved. In addition, the methods used generally ranged from traditional methods (less effective) to modern methods (with varying degrees of effectiveness), so even with this relatively high level of potential demand for contraception and 76.1 % of demand satisfied, the rate of unplanned pregnancy and induced abortions might remain relatively high in this population. However, increasing the potential demand for contraception goes a long way in reducing unmet need for family planning thereby reducing the rate of mistimed or unplanned births, induced abortions and maternal and neonatal morbi-mortality.

Unmet need for family planning, being one of the major indicators for monitoring family planning programmes is therefore supposed to be kept as low as possible and if possible, eliminated if the post-2015 sustainable development target on maternal mortality is to be met. It is but clear that much is still to be done in the domain of objective based sensitization to modify and correct the attitudes of non-users towards contraception.

Among the multiple factors evaluated, only partner’s approval of contraception and discussion of family planning within the couple were found to be significantly associated to unmet need for family planning. A woman whose partner approved of contraception was 0.52 times less likely to have an unmet need than a woman whose husband disapproved (*p* = 0.023). Also, a woman who testified to have discussed about family planning with the husband was 0.39 times less likely to have unmet need than a woman who has never discussed family planning with the husband (*p* = 0.001). These two factors have been proven by many studies to have high significant associations with unmet need for family planning. These two factors have been described in Rural Burkina Faso [14], Kenya [15], and Ethiopia [16] to have significantly protective association with unmet need for family planning. These two factors bring clearly to light the fact that family planning and contraceptive use is a “couple thing”. The liberty a woman has when discussing about family planning and therefore the approval and support of the male counterpart are very important points to look into when carrying out interventions aimed at reducing unmet need for family planning. Even though, a small proportion of women in a union adopt contraceptive practices without the concern of their husbands, the final decider and promoter is generally the man. Sensitization campaigns and counselling in most settings in Cameroon and even during routine general and gynaecological consultations have long targeted the female partner. This has brought some progress in encouraging contraceptive use but if strategies could be brought in to include the man in campaigns and in sessions of early postpartum counselling to these women, it will go a long way in solving issues of unmet need for family planning among these women.

The total number of pregnancy greater than 3 and the number of children alive greater than 5 showed significant exposure to unmet need for family planning in univariate analysis but when checked with potential confounders, the association remains but statistically insignificant. Studies carried out in Africa however have shown significant associations between these factors and unmet need for family planning [17, 18].The fear of side effects stood out as the major reason for the non-use of family planning methods among women with unmet need in this population. These side effects of which the women generally do not know much about. Wrong sensitizers and poor administration of the information about the different family planning methods may explain these misconceptions. Sensitization is therefore supposed to remain corrective of misconceptions with clear presentation of the different methods and their side effects. In 2014 during a systematic review of 51 surveys in the developing world, infrequent sex, fear of side effects or health risks were identified as major reasons for non-use of contraception among women with unmet need [19]. According to a 2009 report, a variety of contraceptive method have been made available and are been sold and given even for free at different levels in Cameroon [20]. Accessibility seems far from being our major problem especially in this highly urbanised setting. Corrective counselling and sensitization is therefore our major way out in surmounting the different reasons women put forth for non-use of contraception.

### Limits and strengths of the study

Given the great role men play in decision making concerning unmet need, this research has a weakness that it did not evaluate unmet need among men. In addition our definition for unmet need was not directly applicable to sexually active women out of union given that other parameters are to be taken into consideration. We used a cross-sectional study model, which can only reveal associations rather than causal relationships between the different variables and the outcomes of interest. Our study did not evaluate the supply and delivery of the family planning services which could be possible factors influencing unmet need. Notwithstanding, our study was designed with proper checks to limit selection bias, lie and measurement bias. The results from this study are therefore internally and externally valid and give a clear picture of the situation on unmet need for family planning in urban Cameroon.

## Conclusion

Following our findings, we conclude that: The prevalence of unmet need for family planning in the district is still very high (20.4 %) and women have more unmet need to space (14.2 %) than to limit (6.2 %). Two major factors still keeping unmet need high in the Biyem-Assi Health District are husband’s disapproval of contraception and non-discussion of family planning within the couple. Major reason for non-use of contraception among women with unmet need for family planning is fear of side effects. The government should improve in counseling strategies while integrating participation of both sexes in sensitization campaigns on family planning and its methods. More emphasis should be put on family planning while counseling not just the woman but the couple in early postpartum. During sensitization campaigns, each method should be presented making sure that the listeners clearly understand the different side effects of the methods. More studies should be carried out in this domain to evaluate the supply and delivery of family planning services in this population while evaluating unmet need for family planning among men and the couples’ unmet need.

## Abbreviations

AOR: Adjusted Odds Ratio
CI: Confidence Interval
MDG: Millennium Development Goals
OR: Odds Ratio
